# The Co-existence of Rheumatoid Arthritis and Granulomatosis With Polyangiitis: Two Cases and Review of the Literature

**DOI:** 10.7759/cureus.17103

**Published:** 2021-08-11

**Authors:** Ahmed S Hassan, Ehizogie Edigin, Axi R Patel, Augustine Manadan

**Affiliations:** 1 Internal Medicine, HealthLinc East Chicago, Indiana, USA; 2 Internal Medicine, John H Stroger Jr Hospital of Cook County, Chicago, USA; 3 Rheumatology, John H Stroger Jr Hospital of Cook County, Chicago, USA

**Keywords:** rheumatoid arthritis, vasculitis, wegener’s, anca, granulomatosis polyangiitis

## Abstract

Granulomatosis with polyangiitis (GPA) and rheumatoid arthritis (RA) have shared features including vasculitis, ocular inflammation, interstitial lung disease, and arthritis but existing evidence indicates they are distinct conditions. Interestingly, the co-existence of GPA and RA has been described in the literature. Herein, we report two cases of GPA developing in patients with underlying RA and examine the relationship between the two conditions.

Two cases of GPA that developed in patients with preexisting RA are described in detail. Additionally, PubMed was searched for articles in English showing an association of RA and GPA using keywords “rheumatoid arthritis, and vasculitis, and Wegener’s, and ANCA, and granulomatosis polyangiitis.”

In addition to our two cases of RA and GPA overlap, 14 reports were identified in PubMed library from 1970 to 2020. Most of the cases were females (14/16, 88%), and had RA as the initial diagnosis (15/16, 94%). The mean age of RA diagnosis was 45.5 years, the mean age of GPA diagnosis was 52 years and the mean interval between both diagnoses was 101 months. Cyclophosphamide and steroid therapy were used in most of the cases.

There are numerous reports of GPA and RA overlap in the literature. GPA should be considered in the differential diagnosis when vasculitis develops in patients with RA.

## Introduction

Granulomatosis with polyangiitis (GPA) is a condition characterized by small vessel vasculitis and granulomas. Areas commonly affected in GPA include the upper airway, lower airway, skin, and kidneys. Rheumatoid arthritis (RA) is chronic autoimmune inflammatory arthritis resulting in joint destruction and systemic features. GPA and RA have shared features including vasculitis, ocular inflammation, interstitial lung disease, and arthritis but existing evidence indicates they are distinct conditions. Interestingly, the co-existence of GPA and RA has been described in the literature [1-11]. Herein, we report two cases of GPA developing in patients with underlying RA and examine the relationship between the two conditions.

Two cases of GPA that developed in patients with preexisting RA are described in detail. Additionally, PubMed was searched for articles in English showing an association of RA and GPA using keywords “rheumatoid arthritis, and vasculitis, and Wegener’s, and ANCA, and granulomatosis polyangiitis.” Clinical details including the timing of diagnoses, age, gender, clinical manifestations, serology, and therapies are reported individually and in aggregate.

## Case presentation

Case 1

**Figure 1:**
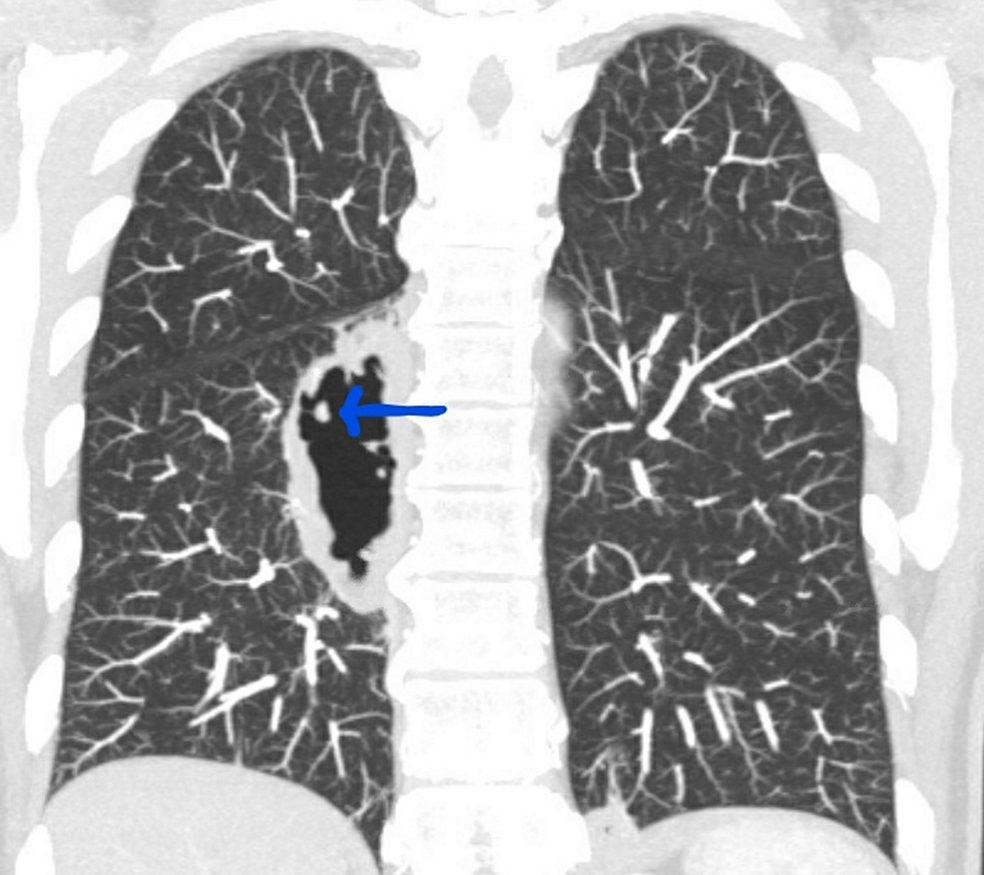
Coronal Reconstruction of Chest Computed Tomography of Case 1 Arrowhead points to cavitary lesion measuring 31 x 78 mm in the right lower lung lobe

A 62-year-old Hispanic female with a past medical history of RA presented with cough, generalized weakness, and a 20-pound weight loss over six months. Her diagnosis of RA was made five years prior based on the bilateral symmetrical synovitis of the metacarpophalangeal (MCP), and proximal interphalangeal joints, a positive rheumatoid factor (RF), and a positive cyclic citrullinated peptide (CCP). Her past medical history was also significant for diabetes, hypertension, and hypothyroidism. Her RA had been well controlled on leflunomide and low-dose prednisone. Initial evaluation showed a white blood cell (WBC) count of 11,300 /mm^3^ and an erythrocyte sedimentation rate (ESR) 64 mm/h. A chest computed tomography (CT) scan showed a new cavitary lesion measuring 31 x 78 mm in the right lower lung lobe, a 16 x 12 mm nodular density in the left lung base with central cavitation, and several ill-defined thin-walled cavitary lesions in both lung apices measuring between 1 and 2 cm in diameter (Figure 1).

Sputum cultures for Mycobacterium tuberculosis were negative. Laboratory testing revealed cytoplasmic anti-neutrophil cytoplasmic antibody (c-ANCA) titer of 1:160 and an elevated proteinase 3 (PR3) antibody of 4.7 units (normal <1 unit). Serial ANCA testing showed a peak titer of 1:320, with PR3 antibody of 26 units. Her first lung biopsy showed focal acute and chronic inflammation, fibrinoid necrosis, and hemorrhage. A second lung biopsy showed fragments of densely fibrous tissue with acute, chronic, and non-caseating granulomatous inflammation consistent with a diagnosis of GPA. No acid-fast bacilli, fungal organisms, or malignant cells were identified. The patient was given two infusions of rituximab 1000 mg intravenously (IV), 15 days apart with methylprednisolone 100 mg IV. Her symptoms as well as her cavitary lung lesions gradually improved. Both RA and GPA remained under good control on maintenance methotrexate (MTX) and prednisone.

Case 2

**Figure 2:**
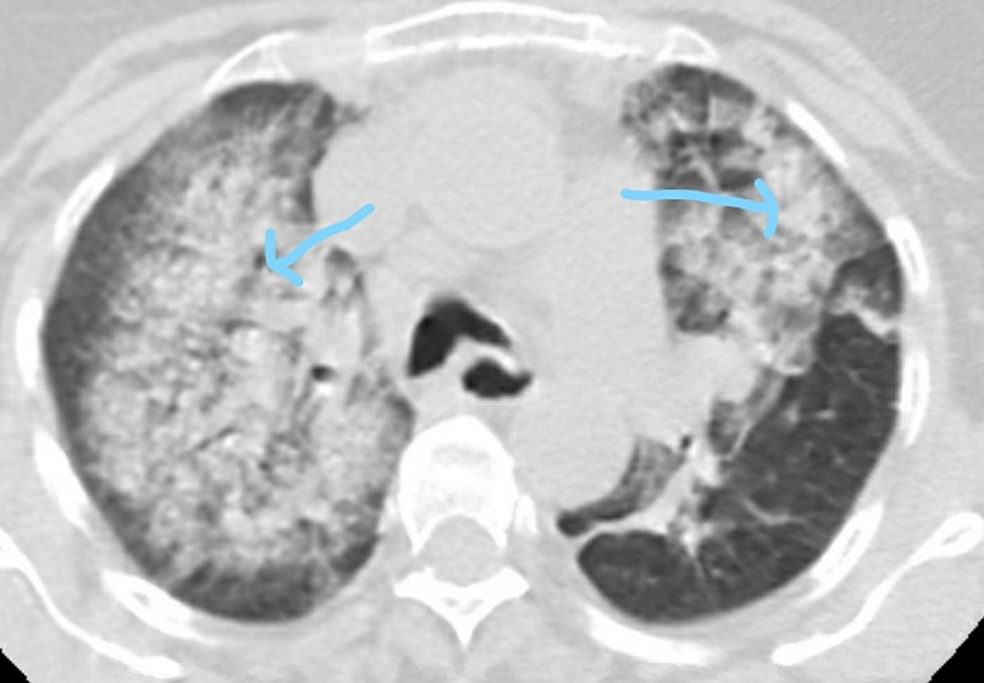
Axial Reconstruction of Chest Computed Tomography of Case 2 Arrowheads point to airspace disease involving the right and left upper lung lobes concerning for diffuse alveolar hemorrhage (DAH)

A 58-year-old Indian female presented with 10 days of dyspnea and hemoptysis. She had a past medical history of RA, diagnosed at age 57 after developing bilateral MCP synovitis, elevated RF of 134 IU/mL, elevated ESR of 116 mm/hour, and hand radiographs showing periarticular osteopenia and bony erosions. Her RA was controlled on MTX, hydroxychloroquine, and low-dose prednisone when she presented with a serum creatinine of 5.5 mg/dL (baseline 1.6 mg/dL) and proteinuria. A chest CT scan showed airspace disease involving the right upper, middle, and lower lobes and left upper lobe concerning diffuse alveolar hemorrhage (DAH) (Figure 2).

Anti-PR3 c-ANCA titer was elevated at 1:320. She was intubated and bronchoalveolar lavage (BAL) confirmed the presence of DAH. Biopsy of the bronchiolar wall showed chronic granulomatous inflammation and fibrosis consistent with GPA. She was given cyclophosphamide 500 mg IV, methylprednisolone IV, and plasmapheresis. She gradually improved and was eventually extubated and discharged home in stable condition. She was discharged on oral cyclophosphamide, which was later switched to azathioprine. Her RA and GPA have remained in remission on azathioprine.

## Discussion

Fourteen reports were identified in the PubMed library from 1970 to 2020. We summarized the clinical and pathological features of those cases in Table 1 (see appendix). 

Including our two cases, most of the cases were females (14/16, 88%), and had RA as the initial diagnosis (15/16, 94%). The mean age of RA diagnosis was 45.5 years, the mean age of GPA diagnosis was 52 years and the mean interval between both diagnoses was 101 months. ANCA and RF antibodies were positive in most of the cases. Cyclophosphamide and steroids were the most common vasculitis treatment regimen.

GPA is one of three known subsets of ANCA-associated vasculitis (AAVs) as outlined in the 2012 revision of the Chapel Hill nomenclature system [12]. GPA is the most well studied epidemiologically among the AAVs with an incidence of around 2 to 15 cases per million inhabitants and an estimated prevalence of up to 160 cases per million inhabitants [13]. RA has an annual incidence of around 40 per 100,000 patients [14]. The coexistence of GPA and RA is thought to be uncommon; however, there have been numerous case reports of GPA diagnosed in patients with RA as described in Table 1. There also have been case reports of RA overlapping with microscopic polyangiitis [15,16].

RA and GPA have some similar features. For example, most patients with GPA will develop arthralgia or arthritis at some point in the disease course [17,18]; however, erosive arthritis is rare, which can likely serve as a key differentiating feature from RA [18]. RA patients can also develop rheumatoid vasculitis (RV), which is often characterized by cutaneous manifestations. These manifestations include purpura, lower extremity skin ulcers, livedo reticularis, digital infarctions, gangrene, and leukocytoclastic vasculitis (LCV) [19].

The etiology of the RA and GPA overlap is not well understood. One possible explanation is that the two conditions could share a common genetic predisposition. Both RA and ANCA-positive GPA were shown to have an increased frequency of a functional polymorphism (620W) of the protein tyrosine phosphatase non-receptor 22 gene (PTPN22) when compared to healthy controls [20]. Mustila et al. found p-ANCA positivity to be independently associated with rapid radiographic destruction in patients with early RA, as well as a significant and independent predictor of RA-associated nephropathy, suggesting a pathogenic role for p-ANCA against myeloperoxidase (MPO) in RA [21]. Finally, Chung et al. concluded the GPA and RA may arise from a similar genetic variation in CTLA4 [22].

Cyclophosphamide and steroids were used in the majority of the reported cases, but we used rituximab successfully in one of our cases. Rituximab is an approved treatment for both RA and GPA so intuitively seems to be a good choice for the overlap condition. There are published case reports of the effective use of rituximab for the treatment of patients with coexisting GPA and RA [23]. 

## Conclusions

Both GPA and RA are rare autoimmune conditions that can rarely co-exist. In addition to our two cases, there are 14 reports of this overlap in the literature. The majority of the cases were females, had RA as the initial diagnosis, and had an average interval between diagnoses of 101 months. Signs of vasculitis especially in the setting of positive ANCAs in patients with RA should prompt consideration of GPA. Rituximab should be studied as a treatment for this overlap condition as it is already an approved treatment for both conditions.

## Appendices

**Table 1:** Cases of GPA and RA overlap Abbreviations: AZA: Azathioprine, BAL: Bronchoalveolar lavage, bx: biopsy; c-ANCA: Cytoplasmic antineutrophil cytoplasmic antibodies, CT: computed tomography, Cyc: Cyclophosphamide, DAH: Diffuse alveolar hemorrhage,  Dx: Diagnosis, ESR: erythrocyte sedimentation rate, F: Female, G: gender, GN: Glomerulonephritis, GPA: Granulomatous polyangiitis, IV: Intravenous, IVIG: Intravenous immunoglobulins, m: Months, M: male,  p-ANCA: Perinuclear antineutrophil cytoplasmic antibodies, PEX, plasmapheresis PO: Per os, PR3: Proteinase 3, RA: Rheumatoid arthritis, RF: Rheumatoid factor, RV: Rheumatoid vasculitis, +: Positive, y: years.

Case report	G	Age at RA Dx	Age at GPA Dx	Initial Dx	Interval between Diagnoses	RA manifestations	GPA manifestations and Pathology	Therapy
Szilasi 2012 [2]	F	40	70	RA	30 y	Hand joint deformities, Periungual RV, + RF, & + CCP	+ PR3 ANCA, Lung bx: chronic granulomatous inflammation, focal fibrosis & obliterative bronchiolitis. Kidney: Crescentic GN with pauci IgM deposition.	Steroids, Cyc, & AZA
Vaishnav 2012 [7]	F	37	37	RA	7 m	Symptoms of RA & + RF	DAH, + cANCA, & pulmonary opacities	Steroids & Cyc
Douglas (1) 2003 [3]	F	35	35	RA	3 m	Polyarthritis, morning stiffness, erosions, & + RF	Saddle nose, hematuria, +PR3 , + c-ANCA, & BAL: DAH with hemosiderin-laden macrophages.	Steroids & Cyc
Douglas (2) 2003 [3]	F	35	55	RA	20 y	Synovitis, erosions, & + RF	Hematuria, + PR3 ANCA, & Lung bx: granulomatous inflammation with capillaritis.	Steroids & Cyc
Chinoy (1) 2002 [5]	M	32	32	RA	2 m	Morning stiffness, synovitis, erosions. nodules. & + RF	Nasal congestion, pharyngitis p-ANCA, Lung: focal necrotizing granuloma.	Steroids, Cyc, & AZA.
Chinoy (2) 2002 [5]	F	41	26	GPA	15 y	Synovitis, erosions, & + RF	Bilateral pulmonary consolidations, + p-ANCA, & Lung bx: focal areas of necrosis and granulation tissue.	Steroids, HCQ, & Cyc.
Guo 2012 [4]	M	60	66	RA	6 y	small joint deformities + RF, & radiographs	Chronic rhinosinusitis, bilateral lung nodule, facial ulcer & bx: granulomatous inflammation and vasculitis	Steroids, Cyc, & IVIG.
Parekh 2010 [10]	F	49	58	RA	9 y	Erosive RA	Hemoptysis, sinusitis, CT chest: bilateral cavitating lung nodules, + PR3 ANCA, & Tracheal bx: extensive infarction and inflammation with necrotizing vasculitis	Steroids & MTX
Ohashi 1991 [6]	F	33	38	RA	5 y	Synovitis, deformity, +RF, & X-rays: joint space narrowing	Perforated nasal septum, saddle nose & nasal cavity bx: arteritis with cellular infiltration, giant cells & epidermoid cells	Steroids & Cyc
Campochiaro 2016 [11]	F	35	55	RA	20 y	Small joint synovitis & + RF	Proteinuria, CT: pulmonary excavated lesions, nasal septum erosions, + PR3 ANCA, skin bx: pauci-immune vasculitis, & neck mass bx: necrotizing granulomatous inflammation.	Steroids, Cyc, & rituximab.
Pai 2008 [1]	F	57	60	RA	3 y	Arthritis, +RF, & + CCP	+ PR3 ANCA & lung bx: fibrosis, inflammation, & necrotizing vasculitis.	MTX & steroids.
Sturrock 1974 [9]	F	40	59	RA	19 y	Polyarthritis, +RF, & erosions	Dyspnea, Piriform fossa bx: mucosal ulceration. Larynx bx: inflammatory granulation tissue & kidney bx: fibrinoid necrosis with crescent formation.	Steroids
Pritchard (1) 1976 [8]	F	45	45	RA	9 m	Polyarthritis & high ESR.	Vasculitic nail bed changes, sinusitis, nasal mucosal atrophy and crusting, pulmonary infiltrates & upper airway bx: granulomatous lesions	Cyc
Pritchard (2) 1976 [8]	F	75	75	RA	5 m	Polyarthritis, nodules. episcleritis, high ESR, & periarticular osteoporosis.	Recurrent otitis media, hemoptysis, sinusitis and nasal bx: necrotizing granuloma consistent with GPA;	Steroids, AZA, & Cyc
Hassan (1) (this work)	F	57	62	RA	5 y	Polyarthritis, +RF, & +CCP	Cavitary lung lesion, + PR3 ANCA, & lung bx: granulomas	Steroids & rituximab
Hassan (2) (this work)	F	57	58	RA	1 y	Polyarthritis, +RF, & erosions	DAH, + PR3 ANCA, & lung bx: granulomas	Steroids, Cyc, PEX

## References

[1] Pai S, Panda M (2008). Limited Wegener's granulomatosis presenting as lung nodules in a patient with rheumatoid arthritis: a case report. Cases J.

[2] Szilasi M, Mátyus J, File I (2012). Association of ANCA-associated vasculitis-rheumatoid arthritis overlap syndrome in four patients: rituximab may be the right choice?. Autoimmunity.

[3] Douglas G, Bird K, Flume P, Silver R, Bolster M (2003). Wegener's granulomatosis in patients with rheumatoid arthritis. J Rheumatol.

[4] Guo Z, Liu Y, Zheng S, Qiu L, Wu J, Xiao T (2013). Chronic unilateral facial ulcer revealing Wegener's granulomatosis in a patient with rheumatoid arthritis. Acta Derm Venereol.

[5] Chinoy H, McKenna F (2002). Wegener's granulomatosis and rheumatoid arthritis overlap. Rheumatology (Oxford).

[6] Ohashi H, Itoh M, Ogawa N (1992). Wegener's granulomatosis in a patient with a rheumatoid arthritis. Intern Med.

[7] Vaishnav KU, Bhatt C, Desai A (2012). Diffuse alveolar haemorrhage in granulomatosis with polyangitis (Wegener's) with coexistent rheumatoid arthritis. BMJ Case Rep.

[8] Pritchard MH (1976). Wegener's granulomatosis presenting as rheumatoid arthritis (two cases). Proc R Soc Med.

[9] Sturrock RD, Ratnesar P (1974). Wegener's granulomatosis occurring in a patient with pre-existing rheumatoid arthritis. Br J Clin Pract.

[10] Parekh K, Ching D, Rahman MU, Stamp LK (2010). Onset of Wegener's granulomatosis during therapy with golimumab for rheumatoid arthritis: a rare adverse event?. Rheumatology (Oxford).

[11] Campochiaro C, Scotti R, Margari S (2016). A case of granulomatosis with polyangiitis: rheumatoid arthritis overlap syndrome presenting as cervical cancer successfully treated with rituximab. Intern Med J.

[12] Jennette JC, Falk RJ, Bacon PA (2013). 2012 Revised International Chapel Hill Consensus Conference Nomenclature of Vasculitides. Arthritis Rheum.

[13] Gibelin A, Maldini C, Mahr A (2011). Epidemiology and etiology of wegener granulomatosis, microscopic polyangiitis, churg-strauss syndrome and goodpasture syndrome: vasculitides with frequent lung involvement. Semin Respir Crit Care Med.

[14] Myasoedova E, Crowson CS, Kremers HM, Therneau TM, Gabriel SE (2010). Is the incidence of rheumatoid arthritis rising?: results from Olmsted County, Minnesota, 1955-2007. Arthritis Rheum.

[15] Palomar R, Castañeda O, Rodrigo E (2005). [Microscopic polyangiitis in a patient with rheumatoid arthritis]. Nefrologia.

[16] Steuer A, Palmer A, Colaco CB (1995). A patient with rheumatoid arthritis and microscopic polyarteritis. Br J Rheumatol.

[17] Hoffman GS, Kerr GS, Leavitt RY (1992). Wegener granulomatosis: an analysis of 158 patients. Ann Intern Med.

[18] Noritake DT, Weiner SR, Bassett LW, Paulus HE, Weisbart R (1987). Rheumatic manifestations of Wegener's granulomatosis. J Rheumatol.

[19] Sayah A, English JC 3rd (2005). Rheumatoid arthritis: a review of the cutaneous manifestations. J Am Acad Dermatol.

[20] Jagiello P, Aries P, Arning L (2005). The PTPN22 620W allele is a risk factor for Wegener's granulomatosis. Arthritis Rheum.

[21] Mustila A, Korpela M, Mustonen J (1997). Perinuclear antineutrophil cytoplasmic antibody in rheumatoid arthritis: a marker of severe disease with associated nephropathy. Arthritis Rheum.

[22] Chung SA, Xie G, Roshandel D (2012). Meta-analysis of genetic polymorphisms in granulomatosis with polyangiitis (Wegener's) reveals shared susceptibility loci with rheumatoid arthritis. Arthritis Rheum.

[23] Yamada A, Sogabe A, Okuda Y (2020). Rituximab used for simultaneous treatment of PR3-ANCA positive vasculitis associated with rheumatoid arthritis: a case report. Clin Case Rep.

